# Protective effects of urocortin 2 against caerulein-induced acute pancreatitis

**DOI:** 10.1371/journal.pone.0217065

**Published:** 2019-05-17

**Authors:** Jingzhen Yuan, Burcu Hasdemir, Tanya Tan, Chintan Chheda, Jean Rivier, Stephen J. Pandol, Aditi Bhargava

**Affiliations:** 1 Cedars-Sinai Medical Center, Los Angeles, CA, United States of America; 2 Veterans Affairs Greater Los Angeles Healthcare System, Department of Medicine, University of California, Los Angeles, Los Angeles, CA, United States of America; 3 The Osher Center for Integrative Medicine, University of California, San Francisco, San Francisco, CA, United States of America; 4 Department of OB/GYN, University of California, San Francisco, San Francisco, CA, United States of America; 5 The Salk Institute, The Clayton Foundation Laboratories for Peptide Biology, La Jolla, CA, United States of America; University of Szeged, HUNGARY

## Abstract

Because little is known about the role of corticotropin-releasing factor (CRF) agonists in regulating responses in pancreatitis, we evaluated the effects of urocortin 2 (UCN2) and stressin1 in caerulein-induced acute pancreatitis (AP) model in rats. Male rats were pretreated with UCN2 or stressin1 for 30 min followed by induction of AP with supraphysiologic doses of caerulein. Serum amylase and lipase activity, pancreatic tissue necrosis, immune cell infiltrate, nuclear factor (NF)-κB activity, trypsin levels, and intracellular Ca^2+^ ([Ca^2+^]_i_) were ascertained. UCN2, but not stressin1 attenuated the severity of AP in rats. UCN2, but not stressin1, reduced serum amylase and lipase activity, cell necrosis and inflammatory cell infiltration in AP. NF-κB activity in pancreatic nuclear extracts increased in AP and UCN2 treatment reduced caerulein-induced increases in NF-κB activity by 42%. UCN2 treatment prevented caerulein-induced degradation of IκB-α in the cytosolic fraction as well as increased levels of p65 subunit of NF-κB in the cytosolic fraction. Pancreatic UCN2 levels decreased in AP compared with saline. UCN2 evoked [Ca^2+^]_i_ responses in primary acinar cells and abolished caerulein-evoked [Ca^2+^]_i_ responses at 0.1nM, and decreased by ~50% at 1.0nM caerulein. UCN2 stimulation resulted in redistribution of a portion of F-actin from the apical to the basolateral pole. UCN2 prevented the massive redistribution of F-actin observed with supraphysiologic doses of caerulein. UCN2, but not stressin1 attenuated severity of an experimental pancreatitis model. The protective effects of UCN2, including anti-inflammatory and anti-necrotic effects involve activation of the CRF_2_ receptor, [Ca^2+^]_i_ signaling, and inhibition of NF-κB activity.

## Introduction

The urocortins (UCN1-3) are members of the corticotropin-releasing factor (CRF) family of neuropeptides [1]. CRF was originally isolated and characterized from the ovine hypothalamus [2] and is a potent stimulator of the secretion of ACTH and β-endorphins [3]. Urocortins are mammalian homologs of Urotensins that were initially discovered in the urophysis gland of the fish [4, 5]. UCNs along with CRF regulate the neuroendocrine responses to stress. UCNs play an important role in regulation of diverse processes such as vascular tone [6–8], cardiac function [9], visceral pain [10], immune cell activation, and gastrointestinal functions [11–16]. The urocortins exert their effects via two known receptors, CRF_1_ and CRF_2_ [17]. UCN1 binds with similar affinity to both receptors, whereas UCN2 and UCN3 bind exclusively to CRF_2_ [18]. Localization of these ligands and receptors has been ascertained in the central nervous system as well as in the peripheral tissues [11, 15, 19–22].

CRF-like immunoreactivity has been described in the endocrine pancreas of several classes of vertebrates that include fish, amphibians, birds, rodents, non-human primates, and humans [3]. In primates, CRF-containing endocrine cells are scattered within the pancreatic islets, whereas in rodents, they are located at the periphery of the islets. CRF-like immunoreactivity was also found to be interspersed between exocrine acinar cells in all species [3]. We have previously shown that CRF is secreted by cultured rat AR42J acinar cells and stimulation of AR42J cells with caerulein results in ~2-fold induction in CRF and *de novo* induction of UCN1 mRNA levels [21]. UCN3 is also present in the islets and thought to be a differentiation marker for β-cells [23]. Both CRF_1_ and CRF_2_ receptors are present in the human and rodent pancreatic islets [23] and CRF_2_ mRNA is reported in pancreatic acinar cells [21].

Caerulein-induced pancreatitis is one of the best-characterized experimental models that involves administration of high (supraphysiologic) doses of caerulein, a cholecystokinin analog. Physiologic doses used cause maximal pancreatic secretion of amylase and lipase [24, 25]. However, supraphysiologic concentrations result in inhibition of pancreatic secretion, elevation of serum enzymes, edema formation, and infiltration of inflammatory cells into the pancreas, and ultimately death of acinar cells [26–28].

In human acinar cells [29], and in rodents, exposure to secretagogues [21, 30], nutrients [31, 32] and radiocontrast [33] agents has been known to induce AP through mechanisms involving intracellular Ca^2+^ signaling, trypsinogen activation, and endoplasmic reticulum (ER) stress. Particularly, NF-κB activation plays a key role in regulating the expression of genes involved in inflammation, cell injury, and cell death; and its role in caerulein-induced pancreatitis has been shown and characterized [34].

In male rats, UCN1, a ligand for both CRF_1_ and CRF_2_ receptors has been shown to increase pancreatic secretory volume and protein secretion as well as to potentiate cholecystokinin-stimulated protein secretion [35]. We have previously shown that UCN1 was induced *de novo* in exocrine pancreatic acinar cells in the murine model of caerulein-induced acute pancreatitis [21]. CRF_2_ receptor mRNA expression was also increased during AP, whereas CRF_1_ receptor mRNA expression was not detected in the acinar cells. UCN1 reduced histologic damage in male, but not female mice. UCN1 also decreased serum amylase levels and reduced ubiquitination of damaged proteins as well as other ER stress responses in a sex-specific manner [21]. However, the role of UCN2 in pancreatitis, the ligand that exclusively binds and activates CRF_2_ receptor, has not been characterized. Here, we report the immunomodulatory effects of UCN2 in pancreas of male rats. We used Stressin1, a synthetic CRF_1_ receptor-specific agonist, to determine the role of CRF_1_ in caerulein-induced model of acute pancreatitis.

## Materials and methods

### Reagents

Stressin1 and mouse (m)UCN2 were provided by Dr. Jean Rivier, The Salk Institute and The Clayton Foundation Laboratories for Peptide Biology, La Jolla, CA 92037, USA. UCN2 was also purchased from Sigma (St. Louis, MO) and from American Peptides (Sunnyvale, CA). Caerulein was obtained Peninsula Laboratories (Belmont, CA). NF-κB p65 Transcription factor assay kit was obtained from Active Motif (Carlsbad, CA). NF-κB, IκB-α, and GAPDH antibodies were obtained from Cell Signaling (Beverly, MA). Ionomycin was purchased from Molecular Probes (Fisher Scientific, CA) and Alexa Fluor 555 Phalloidin was purchased from Cell Signaling Technology (Danvers, MA).

### Animals and animal care guidelines

Male Sprague-Dawley rats (120–150 g) were used in all experiments. The animals were kept in a temperature-(23 ± 2°C) and humidity- (55 ± 5%) controlled room with a 12-hour light/dark cycle. The animals were provided *ad libitum* standard rat chow and tap water. The Institutional Animal Care and Use Committees of the Veterans Affairs Greater Los Angeles Health Care System, Los Angeles and the University of California, San Francisco approved animal care and all procedures, in accordance with the National Institutes of Health guidelines.

### Experimental pancreatitis model

Rats were pre-treated with intraperitoneal (IP) injection of CRF_1_ receptor-specific agonist, stressin1, or CRF_2_ receptor-specific agonist, UCN2, at doses of 10μg/kg body weight. Rats in control group were injected with saline (vehicle). After 30 min, the pre-treated rats received 4 IP injections of caerulein (CR, 20 μg/kg body weight) at 1-hour intervals. Control rats continued to receive saline injections. The rats were euthanized 1 hour after the 4^th^ IP injection of caerulein or saline. Blood was collected in heparinized tubes for serum amylase and lipase determinations. The pancreata were removed, rinsed in ice-cold PBS, and a portion of the tissue was snap frozen in liquid nitrogen and stored at -80°C. A part of the tissue was fixed in 10% paraformaldehyde and paraffin-embedded for histopathological examination.

### Preparation of nuclear extracts and NF-κB DNA binding activity measurement

Nuclear protein extracts were prepared from pancreatic tissue pre-treated saline or mUCN2 for 30 min followed by 1 IP injection of caerulein. Rats were euthanized 30 min later and pancreata were collected. Pancreatic nuclear extracts were prepared using the Active Motif Nuclear Extract Kit (Carlsbad, CA) as per manufacturer’s instructions. NF-κB DNA binding activities in the nuclear protein extracts were measured with ELISA method using Active Motif NF-κB p65 Transcription factor assay kit (Carlsbad, CA) as per the manufacturer’s instructions.

### NF-κB and IκB western blotting

Western blot analysis on cytosolic fraction was performed for NF-κB and IκB-α levels as described previously [36]. Briefly, equal amounts of protein lysates were separated on SDS-PAGE and transferred to nitrocellulose membranes. The membranes were blocked with 5% nonfat dried milk in Tris-buffered saline, pH 7.2 for 2 hours at room temperature followed by overnight incubated with specific primary antibodies NF-κB or IκB-α (1:1000) in Tris-buffered saline containing 3% nonfat dried milk at 4°C. After washes, the membranes were incubated with secondary antibodies conjugated with horseradish peroxidase (1:5000) for 1h at room temperature. Blots were developed using the enhanced chemiluminescence detection kit (Pierce). The bots were subsequently stripped and reprobed with GAPDH (1:1000), which was used as a loading control.

### Enzymatic assays

Serum amylase and lipase levels were determined by using a Hitachi 707 analyzer (Antech Diagnostics, Irvine, CA). Active trypsin in pancreatic tissue was measured using Boc-Gln-Ala-Arg-AMC as a substrate by a fluorogenic assay as described previously [37, 38].

### Histological measurements

Pancreatic tissue was fixed immediately in 10% buffered formaldehyde. Tissue was then embedded in paraffin and 5μm sections were cut and stained with hematoxylin and eosin (H&E). Sections were examined by light microscopy for assessment of parenchymal structure, inflammation, and acinar cell necrosis. Images were acquired using a light microscope (40X objective), and images were captured using all exposures manually set at equal times with a Nikon Eclipse E600 microscope equipped with a digital camera using the SPOT imaging software (Diagnostic Instruments, Sterling Heights, MI).

Quantification of necrosis was performed on H&E stained pancreatic sections. Images from multiple, non-overlapping sections were captured under a high-power field (x400-magnification, 6 to 10 random fields per section). Cells with swollen cytoplasm, loss of plasma membrane integrity, and leakage of organelles into interstitium were considered necrotic. A total of at least 2000 acinar cells were counted on tissue sections from each animal per condition (n = 3-5/group). Inflammatory cell infiltration was also quantified from H&E stained pancreatic sections from 3 rats per group and expressed as the number of inflammatory cells or vacuoles per 100 acinar cells.

### ELISA for UCN2

Rat pancreatic acinar cells were prepared by injecting the pancreas with 1mL of 200 units/mL collagenase and digesting for 25 min at 37°C with agitation. Small clusters of cells were released by agitation and cells were washed 3x by centrifugation in extracellular Ca^2+^ buffer containing 140 mM NaCl, 4.7 mM KCl, 1.13 mM MgCl_2_, 1mM CaCl_2_, 10mM D-glucose, 10 mM HEPES (pH 7.2), and 0.5% BSA. Cluster of acinar cells were treated as follows: a) untreated, b) 0.1nM caerulein (CR), c) 1.0nM CR, d) 100nM UCN2 for 30 minutes at 37°C, pre-treated for 30 min with 100nM UCN2 followed by 30 min treatment with e) 0.1nM CR, or f) 1.0nM CR. Cells were washed in 1x PBS. Protein lysates from primary acinar cells and pancreatic tissues (~150mg) were prepared by homogenizing cells/tissues in 1X PBS containing protease and phosphatase inhibitors (Sigma, St. Louis, MO). The lysate was spun at 10,000 rpm for 15 minutes at 4°C and supernatant (cytoplasmic proteins) was collected and stored at -80KC until use. Total protein was assessed using the DC protein assay (Bio Rad, Hercules, CA). Two concentrations of total protein (30 and 60μg) were used in ELISA assay.

UCN2 ELISA was performed as per manufacturer’s specifications (Aviva Systems Biology, San Diego, CA). Briefly, 30 and 60μg of total protein in 100μL of standard dilution buffer were added into wells of the anti-UCN2 microplate, covered, and incubated at 37°C for 60 minutes. The liquid was discarded by flicking and incubated with 100μL of biotinylated UCN2 detector antibody for 60 minutes at 37°C. The plate was washed 3x with 300μL of 1x wash buffer for 1 minute each. Subsequently, 100μL of 1x Avidin-HRP conjugate was added to the wells and incubated for 30 minutes at 37°C followed by 5x washes. TMB substrate (90μL) was added to each well and the plate was incubated in the dark for approximately 12 minutes to develop the reaction. Stop solution (50μL) was added to each well to terminate the reaction. Standard range used was from 0-50ng/mL of UCN2. Absorbance was read at 450nm and wavelength correction was set to 570nm.

### Phalloidin staining and confocal microscopy

Rat pancreatic acinar cells were prepared as described above. Cluster of acinar cells were treated as follows: a) untreated, b) 1.0nM caerulein (CR), c) 100nM UCN2 for 30 minutes at 37°C, pre-treated for 30 min with 100nM UCN2 followed by 30 min treatment with d) 1.0nM CR. Cells were washed with 1x PBS, embedded in OCT. Sections (20μm) were air dried, washed 3x with 1x PBS, blocked with 1x PBS containing 5% normal horse serum for 30 min at room temperature. Cells were stained with Alex Fluor 555 phalloidin (330nM) as per manufacturer’s specifications. Sections were observed with a Zeiss laser scanning confocal microscope (LSM Meta 510; Carl Zeiss, Thornwood, NY) using an x63 oil objective and images were captured at equal settings.

### Intracellular Ca^2+^ [Ca^2+^]_i_ measurement of primary acinar cells

Pancreas was obtained from adult C57/BL6 mouse and primary acinar cells were prepared by injecting the pancreas with 1mL of 200 units/mL collagenase and digesting for 25 min at 37°C with agitation. Small clusters of cells were released by agitation and cells were washed 3x by centrifugation in extracellular buffer containing 140 mM NaCl, 4.7 mM KCl, 1.13 mM MgCl_2_, 1mM CaCl_2_, 10mM D-glucose, 10 mM HEPES (pH 7.2), and 0.5% BSA. Cells were loaded with Fura-2AM for 30 min at 37°C in buffer containing 1mM CaCl_2_. Cells were washed 3x and ~2,000 acini were seeded on poly D lysine-coated 96-well plates. Cells were sequentially stimulated with UCN2 (10nM or 100nM), caerulein (0.1nM or 1nM) in FlexStation 2 microplate reader (Molecular Devices) using dual wavelengths. Cells were finally stimulated with 1 μM ionomycin 90 seconds after last agonist stimulation, as indicated in the example Ca^2+^ traces to confirm cell viability (n = 6-8/condition). When Fura-2AM-loaded cells are excited at 340nm, emission value increases with increasing Ca^2+^ concentrations. In contrast, when loaded cells are excited at 380nm, Fura-2 signal decreases with increasing Ca^2+^ concentrations. The ratio of 340nm:380nm emissions is reflective of Ca^2+^ concentrations [39]. Agonist-induced peak Ca^2+^ responses were normalized to peak ionomycin-induced responses [40] to obtain changes in Ca^2+^ concentrations. Agonist stimulations included UCN2 at 10-100nM and caerulein at 0.1–1.0nM.

### Statistical analysis

One-way ANOVA followed by Tukey’s multiple comparison was used to analyze data using Prism v8.02 software (GraphPad Software Inc., La Jolla, CA). A p value of p<0.05 was considered statistically significant. Data are shown as mean ± standard error of mean (SEM).

## Results

### UCN2 decreased serum amylase and lipase activity in caerulein-induced pancreatitis

**Fig 1:**
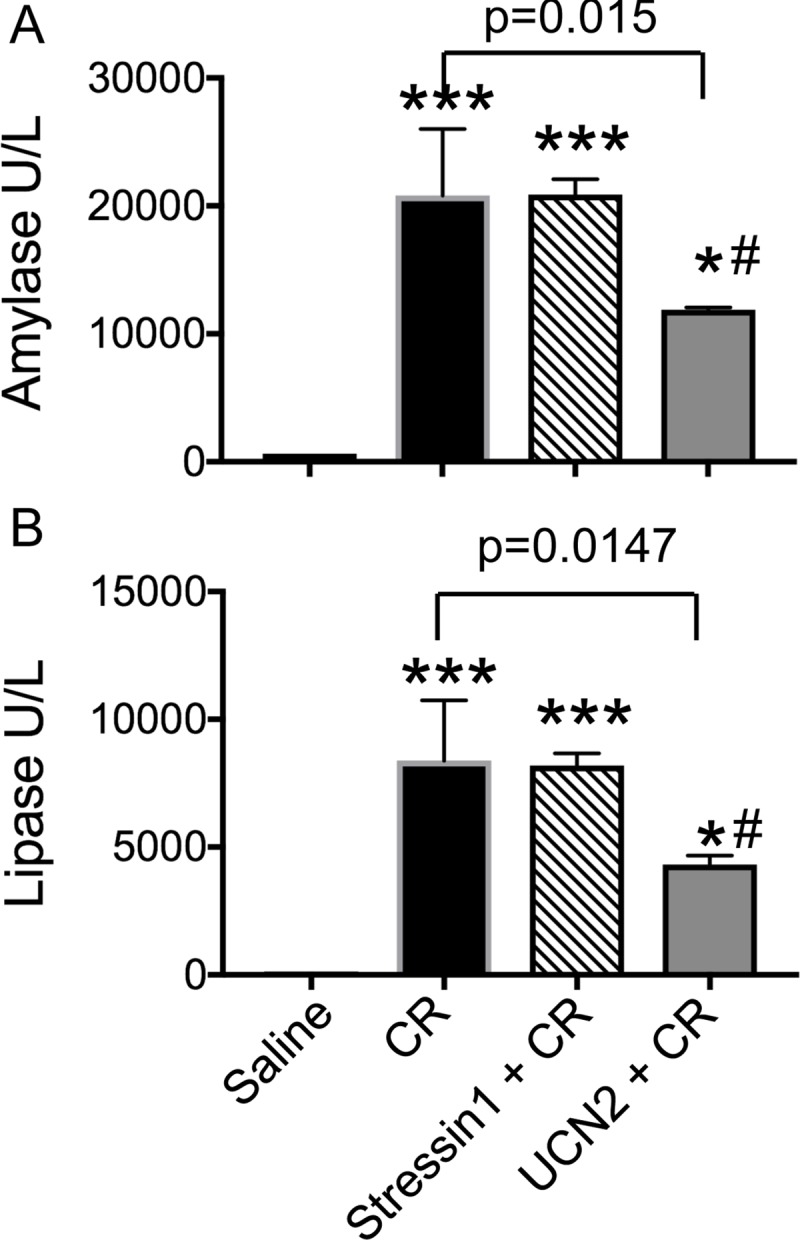
Serum amylase and lipase activity. UCN2 reduced serum (**A**) amylase activity by 43% and (**B**) lipase activity by 48% compared with caerulein treatment. Data are shown as mean ± SD (n = 3), one-way ANOVA and Tukey’s multiple comparisons. CR: caerulein. **Amylase**: ***: p<0.0001 vs. saline; #: p = 0.0038 saline vs. UCN2 + CR; *: p = 0.0145 Stressin1 +CR vs. UCN2 + CR. For **Lipase**: ***: p = 0.0001 vs. saline; #: p = 0.0107 saline vs. UCN2 + CR; *: p = 0.019 Stressin1 +CR vs. UCN2 + CR.

Serum amylase and lipase activities were increased, as expected, following induction of AP with caerulein (Fig 1A and 1B). Pre-treatment of rats with the CRF_2_ receptor agonist, UCN2, significantly reduced caerulein-induced serum amylase and lipase activity by 43% and 48%, respectively. Unlike UCN2, pre-treatment with the CRF_1_ receptor agonist, stressin1, did not change caerulein-induced amylase and lipase activities (Fig 1A and 1B).

### UCN2 decreased acinar cell necrosis

**Fig 2:**
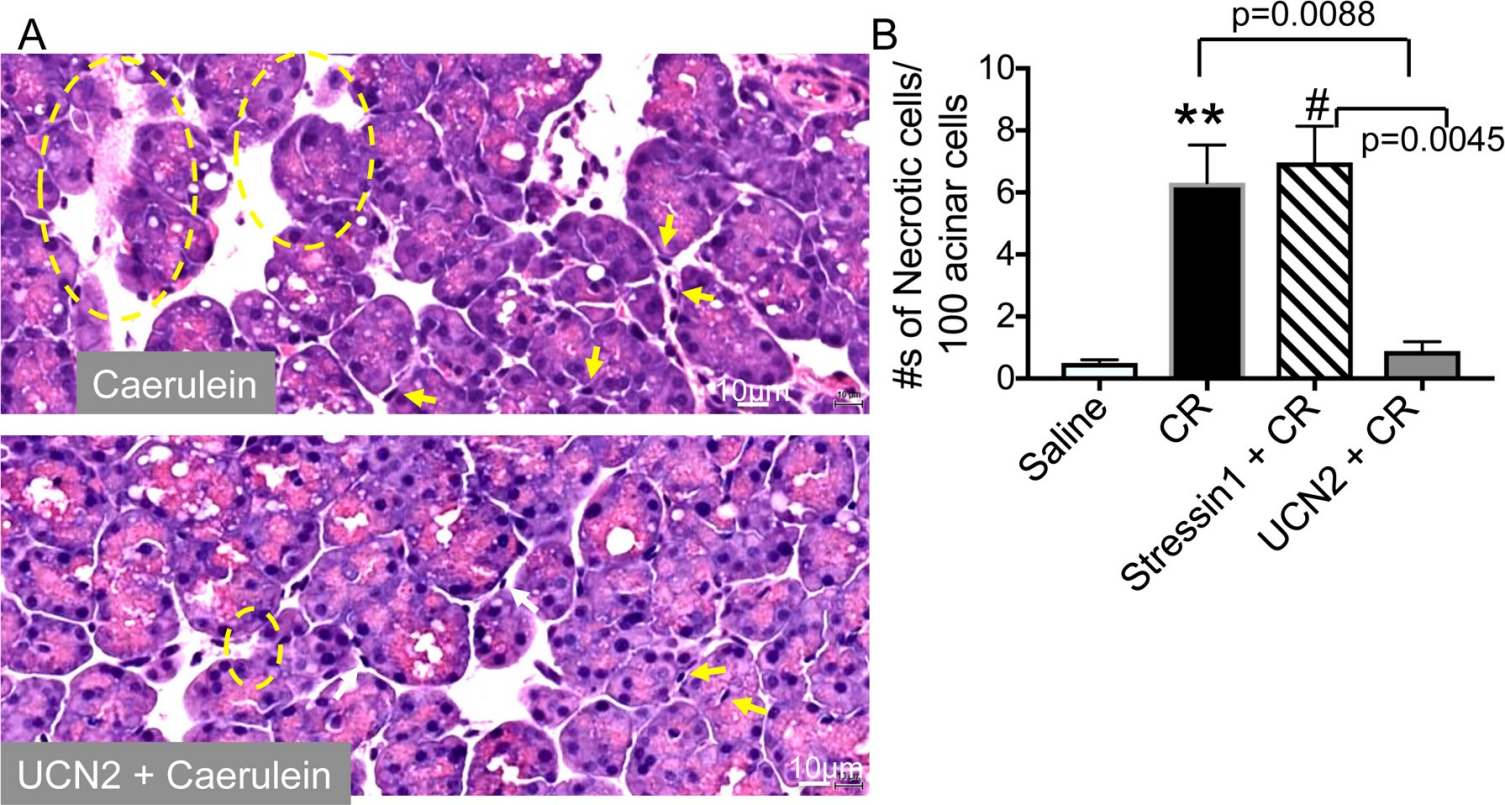
Histopathology and effect of UCN2 on cell necrosis. **(A)** Representative micrographs showing H&E staining of pancreatic tissue sections. Necrotic cells are shown within yellow circles and arrows point to inflammatory cell infiltrates. Scale bar: 20μm. **(B)** Necrosis measurement: Necrosis was assessed on H&E stained pancreatic tissue sections using the following criteria- swollen cytoplasm, loss of plasma membrane integrity, and organelle leakage into interstitium. Data are shown as mean ± SEM (n = 3) for each condition. One-way ANOVA and Tukey’s multiple comparisons. CR: caerulein. **: p = 0.0059 Saline vs. CR; #: p = 0.0030 Saline vs. Stressin1 +CR.

Histological analysis revealed that UCN2-treated pancreatic tissue was well preserved and considerably less necrotic than caerulein-treated tissue (Fig 2A). UCN2 treatment decreased the numbers of necrotic acinar cells by nearly 7-fold compared with caerulein and stressin1 treatments (Fig 2B). Stressin1 treatment had no effect on caerulein-induced cell necrosis (Fig 2B).

### UCN2 decreased infiltrated inflammatory cell numbers

**Fig 3:**
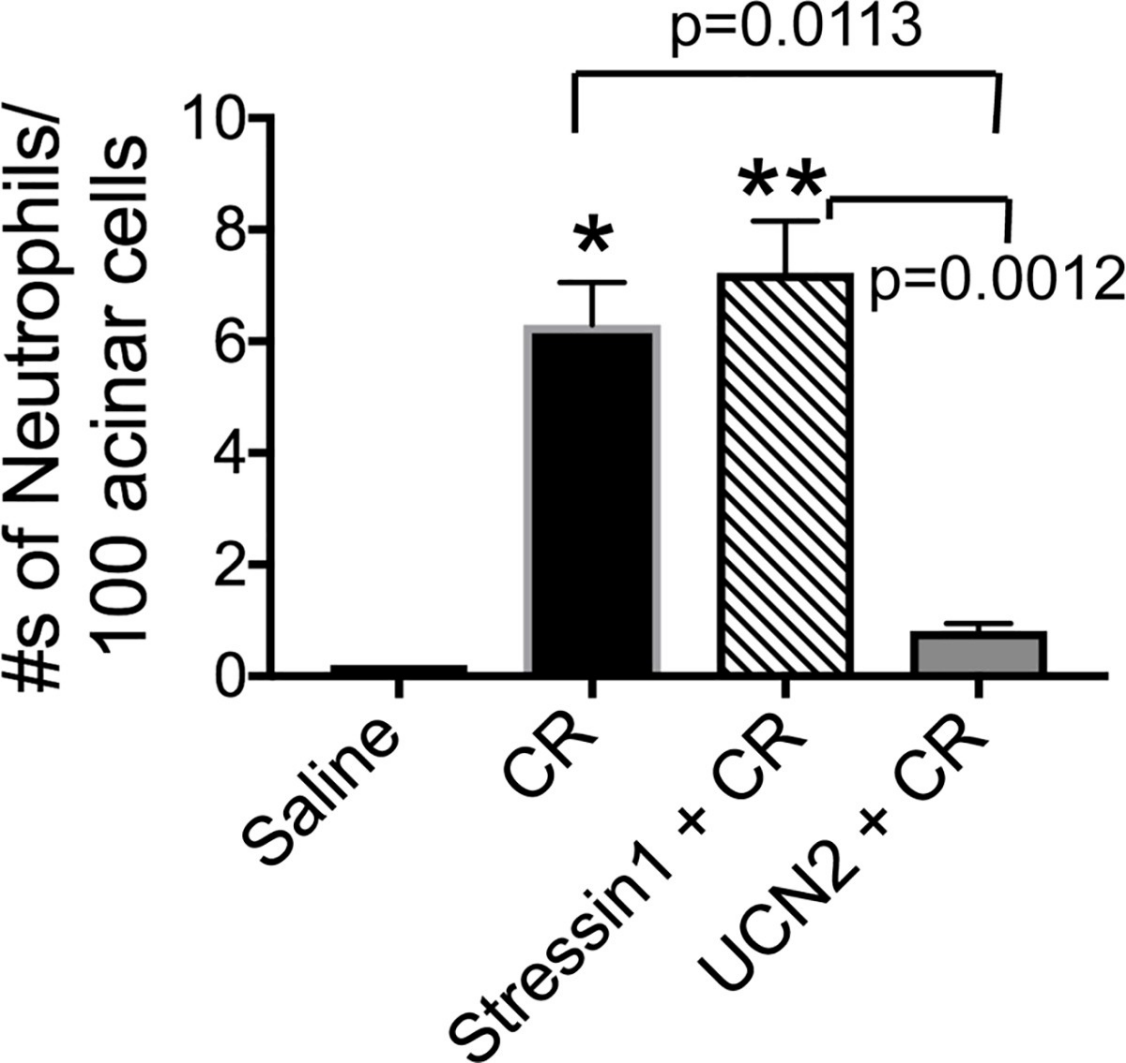
UCN2 reduced neutrophil infiltration. Number of inflammatory cell infiltration (neutrophils) was counted on H&E stained pancreatic tissue sections and expressed as percentage of total acinar cells. Data are shown as mean ± SEM (n = 3) for each condition. One-way ANOVA and Tukey’s multiple comparisons. CR: caerulein. *: p = 0.0112 Saline vs. CR; **: p = 0.0012 Saline vs. Stressin1 +CR.

Infiltration of polymorphonuclear cells is seen during induction of acute pancreatitis. As expected, caerulein treatment markedly increased numbers of infiltrated immune cells in the pancreas, whereas UCN2 treatment decreased immune cell infiltration by nearly 7-fold (Fig 3). Stressin1 did not alter the numbers of immune cells (Fig 3). These results indicate that activation of the CRF_2_ receptors, but not the CRF_1_ receptors, attenuates pancreatic responses.

### UCN2 levels decreased after acute pancreatitis induction

**Fig 4:**
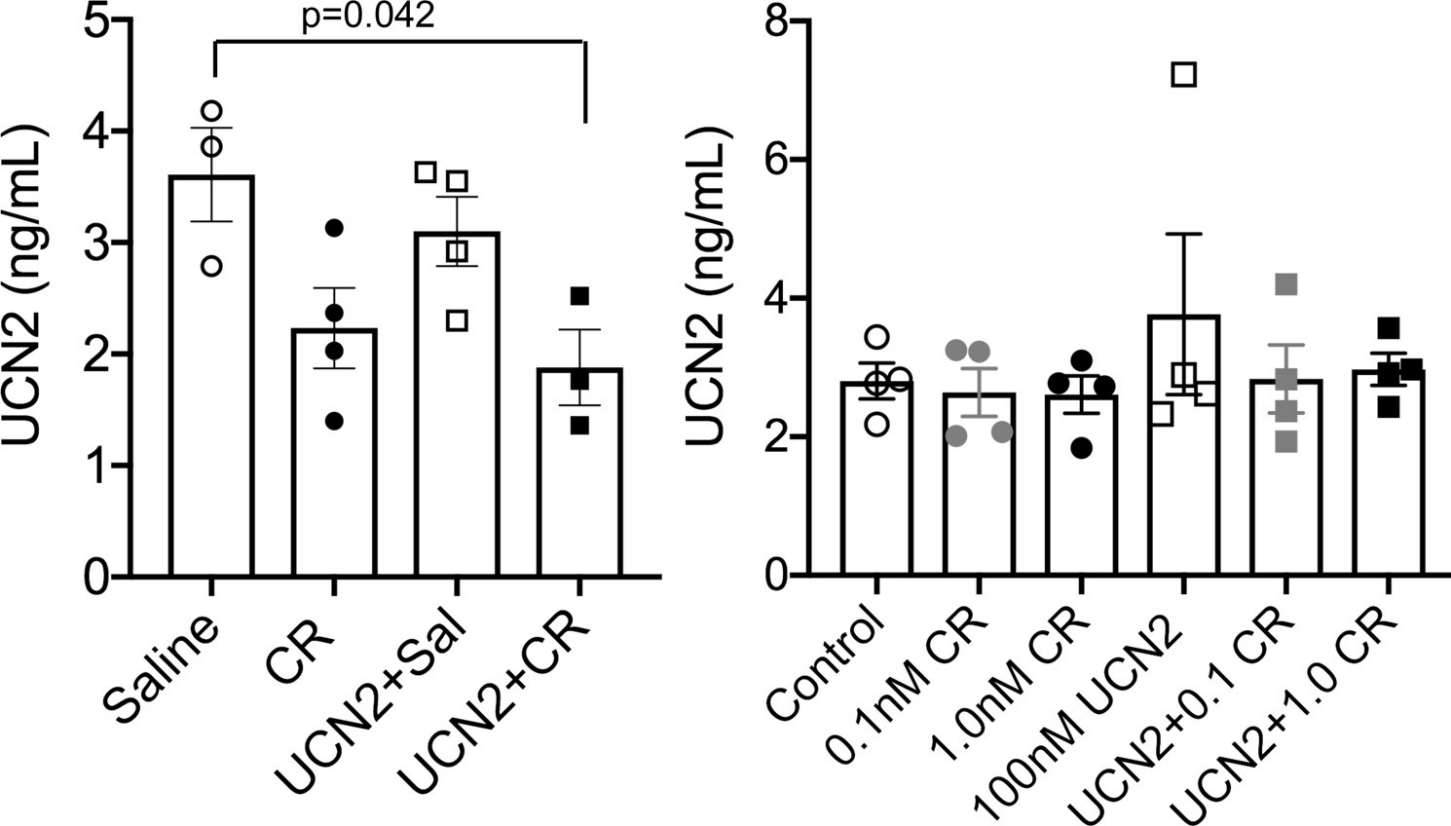
Loss of UCN2 expression in caerulein-induced pancreatitis. UCN2 levels decreased in pancreatic lysates from UCN2 + caerulein-treated rats compared with saline-treated rats (p = 0.042), 1-way ANOVA, whereas UCN2 levels did not change in primary acinar cells.

Since UCN2 administration had anti-inflammatory actions, we next ascertained if caerulein altered UCN2 levels in the pancreatic lysates as well primary acinar cells using ELISA. Compared with saline-treated rats, caerulein treatment decreased UCN2 in the pancreas. Pre-treatment with UCN2 resulted in further depression of endogenous UCN2 levels in the pancreas even in the face of caerulein treatment (Fig 4). In contrast, treatment of primary acinar cells with caerulein or UCN2, did not change UCN2 levels (Fig 4), suggesting that paracrine input is needed for this change to occur.

### UCN2 inhibited activation of NF-κB

**Fig 5:**
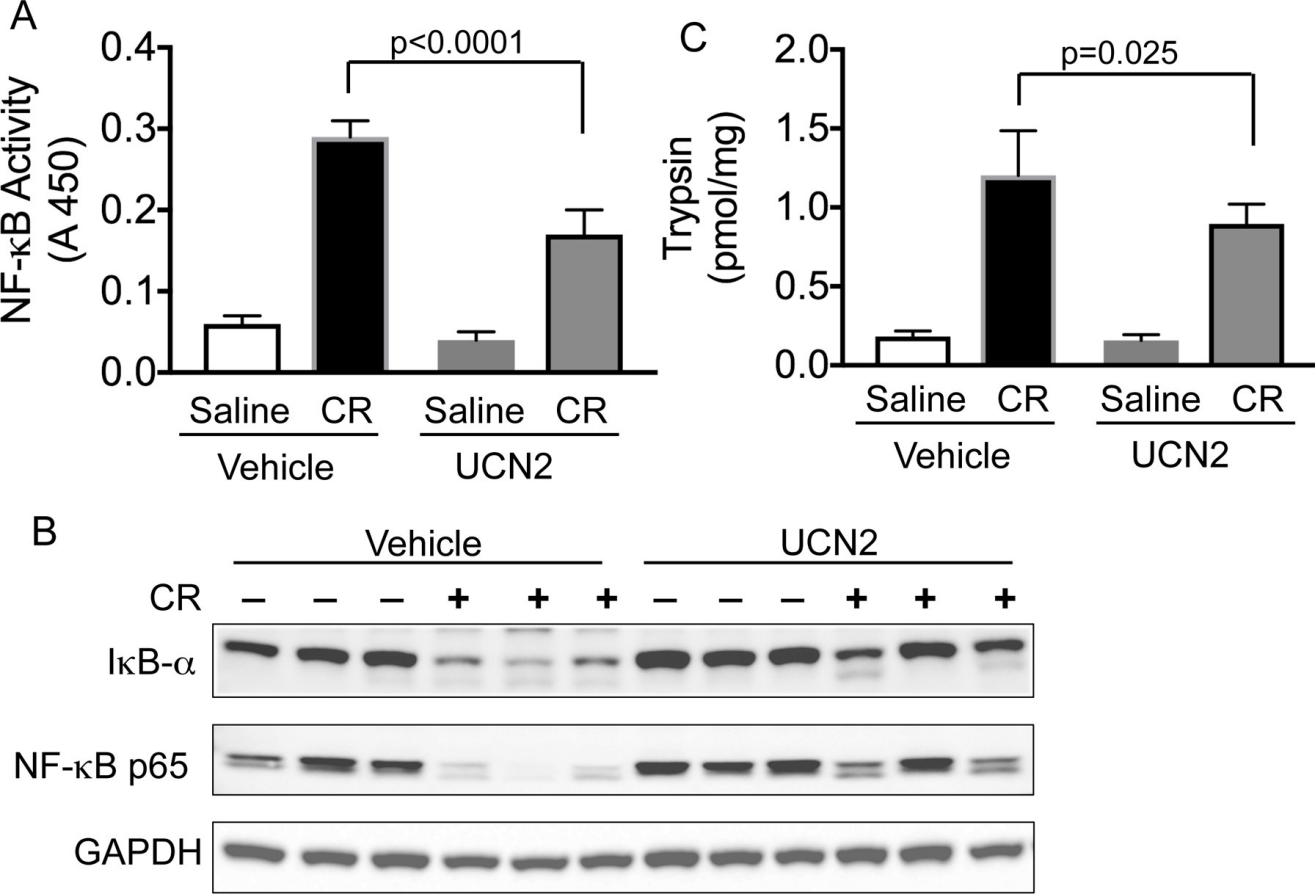
Effect of UCN2 on NF-κB and trypsin activity and IκB degradation. Rats were pre-treated with saline or UCN2 for 30 min followed by treatment with caerulein for 1 hour. (**A**) NF-κB DNA binding activities were measured in the pancreatic nuclear extracts using ELISA. UCN2 treatment decreased NF-κB by nearly 42% in the nuclear extracts. (**B**) Western blot analysis showed decreased IκB-α and NF-κB levels in pancreatic tissue cytosolic extract after caerulein treatment compared with saline controls, suggesting either increased degradation IκB-α or increased translocation of NF-κB from the cytosol to the nucleus. UCN2 treatment prevented caerulein-induced changes in IκB-α degradation and NF-κB nuclear translocation. GAPDH, a housekeeping protein was used as a loading control (n = 3/condition). (**C**) Trypsin activity was increased after caerulein treatment and UCN2 treatment had a modest (15%) effect on trypsin activity. Values are means ± SEM (n = 5). One-way ANOVA and Tukey’s multiple comparisons. CR: caerulein.

Translocation of NF-κB subunits from the cytoplasmic compartment to the nucleus results in transcriptional activation of a number of pro-inflammatory genes in pancreatic acini [34]. Next, we ascertained if UCN2 mediated its anti-inflammatory effect in AP via the NF-κB signaling pathway. As expected, caerulein treatment resulted in increased NF-κB activity in the nuclear fraction, whereas UCN2 treatment decreased NF-κB by nearly 42% (Fig 5A).

### UCN2 modulated trypsin activity

Trypsin activity and levels are increased in pancreatitis. As expected, caerulein treatment resulted in a ~6.6-fold increase in trypsin activity in pancreatic lysates, whereas pre-treatment with UCN2 resulted in ~5.6-fold increase in trypsin activity (Fig 5C). UCN2 treatment caused a modest (15%), but significant decrease in trypsin activity, thereby suggesting that UCN2-mediated mechanisms are largely independent of trypsinogen activation, at least in the acute phase.

### UCN2 modulated intracellular Ca^2+^ [Ca^2+^]_i_ levels in primary acinar cells

**Fig 6:**
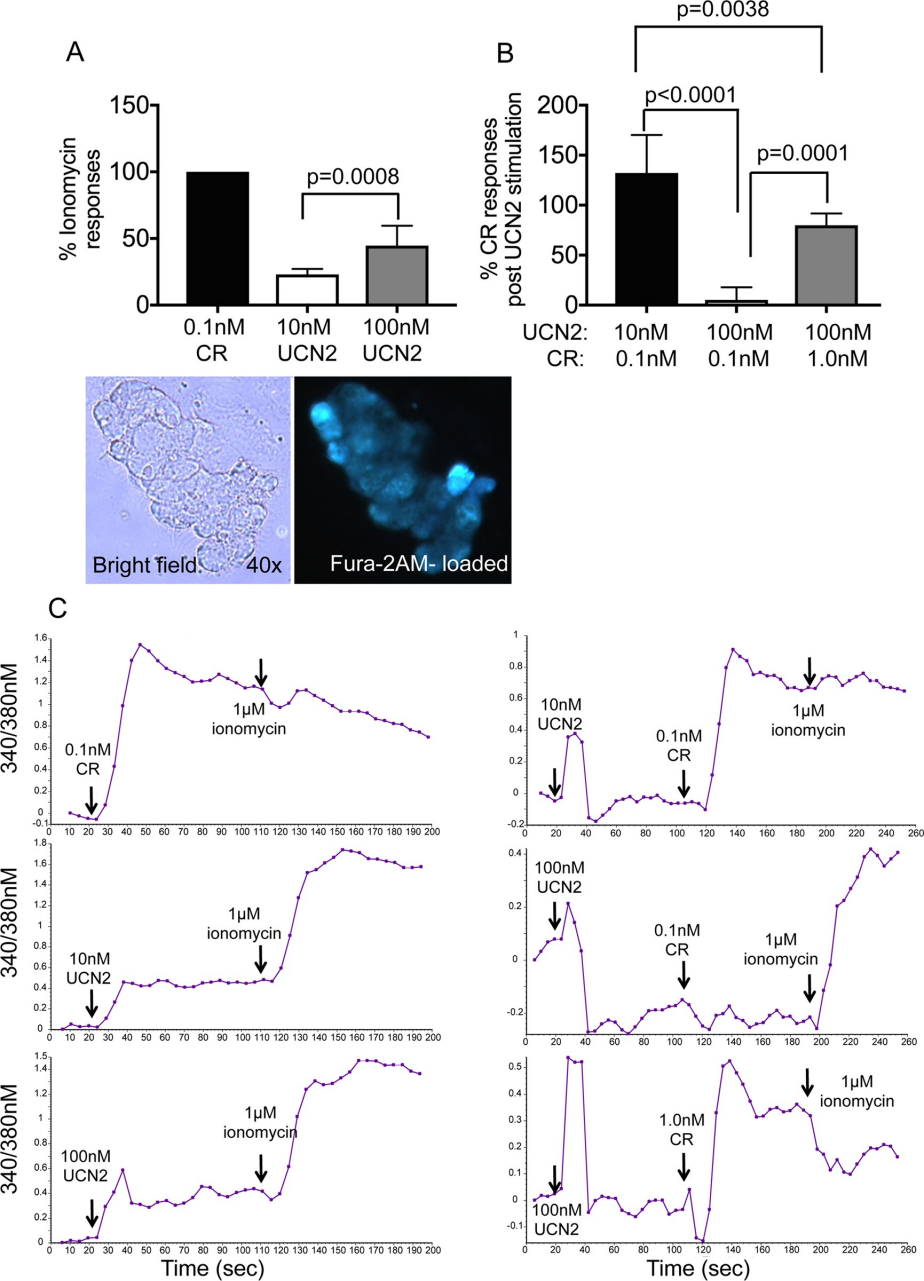
UCN2 evokes Ca^2+^ transients in primary acinar cells. Primary acinar cells were loaded with Fura2AM and stimulated with secretagogues as shown. (**A**) Peak [Ca^2+^]_i_ increased in response to stimulations with 0.1nM caerulein (CR), 10nM, and 100nM UCN2. Ca^2+^ peaks were normalized to ionomycin responses. Stimulation with UCN2 showed dose-dependent increases in Ca^2+^ responses. Lower panel shows a cluster of Fura2AM-loaded acini. (**B**) Primary acinar cells were first stimulated with 10nM or 100nM of UCN2 followed by stimulation with 0.1nM or 1.0nM CR as shown. 100nM of UCN2 completely abrogated CR to induce Ca^2+^ peak responses at 0.1nM and reduced Ca^2+^ peak responses by 50% at 1.0nM, whereas 10nM UCN2 was not effective at modulating CR-induced Ca^2+^ responses. (**C**) Representative traces of Ca^2+^ peaks generated after stimulation with CR, UCN2, and ionomycin. N = 6-8/condition. One-way ANOVA and Tukey’s multiple comparisons.

One mechanism by which caerulein mediates its detrimental effects on secretion are by increasing [Ca^2+^]_i_ to levels that if remained unchecked can lead to organelle damage and cell death via mechanisms that involve mitochondrial depolarization. In primary acinar cells, UCN2 treatment evoked [Ca^2+^]_i_ responses at concentrations of 10nM and 100nM (Fig 6A–6C). Interestingly, 100nM UCN2 prevented increases in [Ca^2+^]_i_ at 0.1nM caerulein and reduced responses by 50% at 1.0nM caerulein, a dose that results in prolonged and sustained increases in [Ca^2+^]_i_ and pancreatitis responses (Fig 6B and 6C). These results indicate that the actions of UCN2 and caerulein differ with respect to [Ca^2+^]_i_ responses, although the reasons for these differences are not revealed here.

### UCN2 treatment prevented caerulein-induced redistribution of F-actin filaments

**Fig 7:**
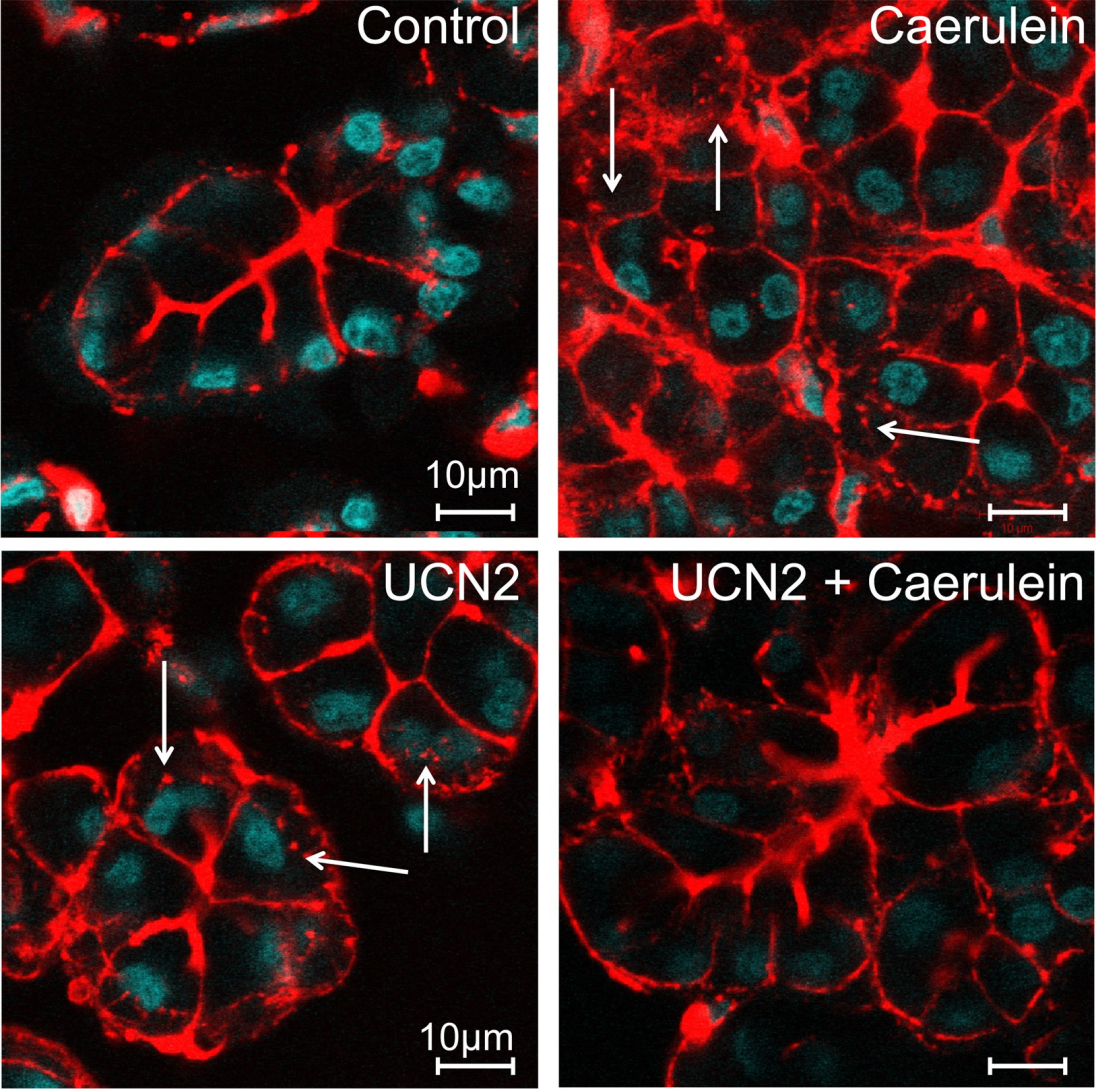
UCN2 prevents F-actin redistribution and over secretion in the face of caerulein. Pancreatic primary acinar cells were stained with Alexa Fluro 555 phalloidin to visualize F-actin bundles. (**A**) in unstimulated acini, F-actin was primarily seen at the apical poles. (**B**) stimulation with 1.0nM caerulein or (**C**) 100nM UCN2 for 30 minutes resulted in robust redistribution of F-actin from the apical to the basolateral pole, along with presence of secretory granules near the basolateral surface (arrows). (**D**) Pre-treatment with 100nM UCN2 followed by 1.0nM caerulein for 30 min restored apical F-actin expression.

Supraphysiologic doses of caerulein are known to cause redistribution of F-actin from the apical to the basolateral pole [41]. We next stimulated primary rat acinar cells with caerulein or UCN2. In unstimulated acinar cells, F-actin was mostly apical in location (Fig 7A), whereas stimulation with supraphysiologic doses of caerulein (1.0nM) resulted in marked redistribution of F-actin from the apical to the basolateral membranes (Fig 7B, arrows). Our results here show that stimulation with 100nM UCN2 caused partial redistribution of F-actin from the apical to basolateral area of the cell (Fig 7C, arrows) and prevented the massive redistribution caused by caerulein (Fig 7D).

## Discussion

Cellular stress responses are key for maintaining normal cellular function and homeostasis. Components of the CRF family of neuropeptide hormones that include UCNs have been shown to modulate immune function, cellular signaling [11–16] as well as secretion [21]. Here, we show that UCN2 ameliorated caerulein-induced pancreatitis measured by reductions in levels of serum amylase and lipase; and tissue measurements of cell necrosis, immune cell infiltration, as well as by inhibition of NF-κB activation. UCN2 treatment prevented caerulein-induced degradation IκB-α as well as nuclear translocation of NF-κB. Finally, UCN2 was found to be a secretagogue that evoked [Ca^2+^]_i_ responses and well as redistributed F-actin in acinar cells.

UCN2 is potent vasodilator and one possible mechanism by which it could influence pancreatic function is by increasing blood flow to the organ, which is known to be compromised in pancreatitis [42]. UCN1, a closely related peptide and which interacts with both CRF_1_ and CRF_2_ receptors has also been shown to reduce serum amylase and inflammation in caerulein-induced pancreatitis model in mice [21]. Interestingly, stressin1, a CRF_1_ receptor agonist did not have effects on caerulein-induced pancreatitis suggesting that the protective effects of both UCN1 and UCN2 are likely mediated via CRF_2_ receptor. In contrast to UCN1 levels, which have been shown to increase in caerulein-induced pancreatitis [21], in this study, UCN2 levels decreased. Taken together, these data suggest that an interplay and balance between different UCNs is needed to fine tune inflammatory responses in pancreatitis.

Cytokines and chemokines play an important role in mediating inflammation. Translocation of the NF-κB from the cytoplasm to the nucleus results in activation of multiple pro-inflammatory genes and increases in circulating levels of cytokines in pancreatitis models [34]. Prevention of NF-κB translocation to the nucleus has a direct effect on TNF-α [43] and other cytokines levels [34]. We found that treatment with UCN2 decreased nuclear NF-κB activity, whereas stressin1 did not. Furthermore, while as expected, caerulein treatment resulted in degradation of cytosolic IκB-α as well decreased expression of NF-κB, UCN2 treatment prevented caerulein-induced degradation of IκB-α, as well as nuclear translocation of NF-κB. These data further suggests that UCN2 actions in the pancreas might be mediated by CRF_2_ receptor. In agreement with this notion, CRF_2_ receptor knockout mice have more exacerbated histological damage than wild-type controls [21].

Intra-pancreatic activation of trypsinogen is involved in pancreas injury and pancreatitis. Increased pancreatic trypsin levels are seen in animal models of pancreatitis [44]. While caerulein treatment increased trypsin activity in the pancreas in our studies, UCN2 treatment had a modest effect on trypsin activity, suggesting that UCN2-mediated mechanisms are only partly involved in trypsinogen pathways.

Secretion from the pancreatic acinar cells is tightly regulated by intracellular concentrations of Ca^2+^ as well as redistribution of F-actin. However, sustained increases in [Ca^2+^]_i_ that are a predominant feature of caerulein-induced pancreatitis are detrimental, leading to organelle damage, inhibited secretion, and can ultimately result in cell death [45]. We found that UCN2 also evokes [Ca^2+^]_i_ responses in primary acinar cells as well as redistributes F-actin from the apical to the basolateral surface. Interestingly, UCN2 treatment blocked caerulein-stimulated Ca^2+^ responses in cells treated with more physiologic concentrations of caerulein. At high doses of caerulein that cause pancreatitis like symptoms, UCN2 reduced caerulein-evoked [Ca^2+^]_i_ by 50% and also prevented the massive redistribution of F-actin. While UCN2 is known to increase both cAMP and Ca^2+^ levels in other cell types [40], the mechanisms of these effects of UCN2 on [Ca^2+^]_i_ and F-actin redistribution, both alone and with caerulein in acinar cells remain to be elucidated.

In conclusion, we show that UCN2 attenuated experimental pancreatitis; its protective effects including anti-inflammatory and anti-necrotic effects are potentially due the effect of UNC2 on activation of NF-κB pathway [46]. UCN2 also mediates its protective effects via F-actin redistribution and modulation of intracellular [Ca^2+^]_i_ levels.
